# Lactate as a Signaling Molecule That Regulates Exercise-Induced Adaptations

**DOI:** 10.3390/biology5040038

**Published:** 2016-10-08

**Authors:** Minas Nalbandian, Masaki Takeda

**Affiliations:** 1 Graduate School of Sports and Health Science, Doshisha University, Kyoto 610-0394, Japan; 2 Faculty of Sports and Health Science, Doshisha University, Kyoto 610-0394, Japan; mtakeda@mail.doshisha.ac.jp

**Keywords:** lactate, signaling molecule, HIF-1, PGC-1 alpha

## Abstract

Lactate (or its protonated form: lactic acid) has been studied by many exercise scientists. The lactate paradigm has been in constant change since lactate was first discovered in 1780. For many years, it was unfairly seen as primarily responsible for muscular fatigue during exercise and a waste product of glycolysis. The status of lactate has slowly changed to an energy source, and in the last two decades new evidence suggests that lactate may play a much bigger role than was previously believed: many adaptations to exercise may be mediated in some way by lactate. The mechanisms behind these adaptations are yet to be understood. The aim of this review is to present the state of lactate science, focusing on how this molecule may mediate exercise-induced adaptations.

## 1. Introduction

Recently, new data suggest that lactate may be playing an important role in the communication between cells and tissues. This concept is relatively new, as well as the idea of lactate being a useful molecule. For a long time, lactate has been argued to mainly cause fatigue and be a waste product of glycolysis. Currently, it is accepted that lactate is not responsible for fatigue, and that covertly, it is an efficient energy source and plays an important role in exercise-mediated adaptations.

The aim of this review is to provide a short introduction to the lactate paradigms and then discuss the possible signaling pathways where lactate may be playing a role (mainly in muscle fibers, fat, and the brain). The first segment will introduce the old lactate paradigm; this will be followed by a presentation of the new paradigm. Then, in the following sections, the possible role of lactate as a signaling molecule will be discussed. Finally, in order to stimulate future research, some unknowns will be presented.

## 2. Old Lactate Paradigm

A detailed discussion of lactate history is beyond the scope of this review; nonetheless, for a better understanding, the traditional role of lactate will briefly be introduced. Readers interested in lactate history should read the review by Philp et al. [1].

Lactate has been a molecule that has generated a great deal of polemics in the history of sports physiology. It was discovered in 1780 when Carl Wilhelm Scheele obtained it from milk [2]. In 1808, Jöns Jacob Berzelius reported that lactate was produced in muscles during exercise, and its structure was established in 1873 by Johannes Wislicenus [1].

During the twentieth century, lactate has been seen as a waste product of glycolysis and its production was associated with muscular fatigue. Early studies showed that lactate increased with exercise in an intensity-dependent manner; therefore, it was strongly correlated with fatigue levels. The predominant theory was that lactic acid was produced from glycolysis: lactic acid is the acid form of lactate, which means it can donate a proton by releasing a hydrogen, in which case it will be converted into lactate (base form). At body pH, lactic acid, which has a low pK (i.e., acid dissociation constant), dissociates immediately into lactate and hydrogen (H^+^), therefore causing a decrease in the pH and producing muscular acidosis, which is even now argued as one of the possible causes of fatigue during high-intensity exercise [3,4,5]. This paradigm presented lactate as a useless molecule that lacks function in the organism.

## 3. New Lactate Paradigm

Since the 1980s, the lactate paradigm has drastically changed, starting with the acceptance that lactate is not responsible for acidosis. This change is clearly reviewed in Robergs et al. [6]. According to this theory, all the intermediate acids of glycolysis have a low pK (included lactic acid); therefore, at body pH levels they all exist in their base form. Furthermore, when the first “intermediate acid” in glycolysis (3-phosphoglycerate) is produced, there is no proton present to be released. As a consequence, pyruvic acid (as well as lactic acid) production is extremely low; instead, pyruvate and lactate are produced, and H^+^ production hence does not come from lactate. The acidosis associated with exercise has been argued to be induced by the increased H^+^ production from the ATP (adenosine triphosphate) hydrolysis (Equation (1)). Conversely, lactate production, transport, and metabolism may be an efficient way to control pH variations during exercise (ut infra). Despite the fact that lactate does not contribute to a decrease in pH, it has been argued to cause fatigue by itself. Hogan introduced this idea in 1995 [7]. Later studies [8,9] have suggested that lactate downregulates the activity of the glycolytic enzyme phosphofructokinase (PFK), therefore reducing energy production (this will be later discussed).

ATP + H_2_O ⟸======⟹ ADP + P*_i_* + H^+^ ATPase
(1)

Lactate as an energy source has also been studied, and it is considered as both an immediate energy source and a contributor augmenting energy reserves. Muscle fibers are classified into two main types: glycolytic and oxidative fibers. During exercise, the demand for energy is increased and glycolytic fibers cover this demand by catabolizing glucose, which brings, as a consequence, increases in pyruvate production. When pyruvate production exceeds the glycolytic fiber capacity to consume pyruvate, it is reduced to lactate, a reaction that is mediated by the action of the enzyme lactate dehydrogenase A (LDHA). After being produced, lactate is co-transported with an H^+^ molecule (in a 1:1 molecule ratio) out of the cell by a simple diffusion facilitated by a monocarboxylate transporter 4 (MCT4). Once in the interstitial space or in the blood flow, it can be co-transported with one H^+^ molecule by a monocarboxylate transporter 1 (MCT1) into an oxidative fiber where it can be metabolized (it is important to note that lactate may also be shuttled to the heart where it is used as an energy source, to the liver for glucose synthesis, and to other places such as fat and the brain, which will be discussed later). Once in the oxidative fiber, lactate is converted into pyruvate by the action of lactate dehydrogenase B (LDHB), and pyruvate is finally aerobically metabolized in the mitochondria. This theory was introduced by Brooks in 1986 under the name of the “lactate shuttle theory” [10]. From this perspective, lactate transport provides an effective way of regulating the accumulation of H^+^ in glycolytic fibers. During exercise, in glycolytic fibers, H^+^-lactate co-transport is responsible for approximately 70%–75% of the release of H^+^ [11]; therefore, the lactate export helps to protect glycolytic fibers from intracellular acidosis. Furthermore, when lactate is produced from pyruvate, it consumes two additional hydrogen molecules (one H and one H^+^) and releases one molecule of NAD (nicotinamide adenine dinucleotide), which is needed for the glycolysis. In the oxidative fibers, the opposite reaction takes place, producing pyruvate and NADH (both needed for oxidative phosphorylation) from lactate and NAD (Equation (2)). From this perspective, lactate production (in glycolytic fibers) and reduction into pyruvate (oxidative fibers) change the cell redox balance, a process that has particular importance during high-intensity exercise [12]. It should also be noted that lactate aerobic metabolism has been suggested to be an important energy source during high-intensity exercise [13,14]; thus, this process is of relevance during that exercise modality.

<<=LDHB Pyruvate + NADH^+^ + H^+^ ⟸======⟹ Lactate + NAD LDHA=>>(2)

In addition, lactate has been traditionally seen as a precursor molecule for glycogenesis and gluconeogenesis in the liver: lactate produced in the muscle is transported through the blood to the liver, where it is converted into pyruvate, and pyruvate is then used for glucose synthesis (gluconeogenesis). Moreover, other studies have suggested that glucose may also be synthesized from lactate in the muscles [15,16], being of particular relevance for the restoration of muscular glycogen after exercise [17,18].

The lactate shuttle theory brought to the field a new research focus: lactate as a signaling molecule. Lactate is a molecule that is transported between cells and tissues, and this molecular exchange may be affecting the places targeted by lactate in many ways. The following sections of this review will focus on how the signals carried by lactate are interpreted by the target tissues, which adaptations may be induced, and which mechanisms underlie these adaptations.

## 4. HIF-1 Mediates Lactate Related Adaptations in Hypoxia

In glycolytic muscle fibers, the transcription factor hypoxia inducible factor-1 (HIF-1) activity is increased, particularly in response to high-intensity exercise [19]. This incremented activity generates many adaptations that include an increase in the anaerobic glycolytic capacity [20,21]. HIF-1 mediated adaptations influence lactate production, transport, and metabolism; furthermore, there are potential reciprocal control mechanisms by lactate on HIF-1 activity.

HIF-1 is recognized for being the master regulator of oxygen homeostasis [20,21,22], and it has been reported to be more active in glycolytic than in oxidative fibers [23]. HIF-1 is a heterodimeric transcription factor that has two sub-units: HIF-1 alpha (oxygen-regulated subunit) and HIF-1beta. After its synthesis, HIF-1 alpha is hydroxylated on proline residues (Pro402 and Pro564) by prolyl hydroxylase 1-3 (PHD1-3). This allows the recognition and ubiquitination by the von Hippel–Lindau ubiquitin ligase E3 (VHL E3), starting a process that ends with the degradation of the protein complex by the 26s proteasome. During hypoxia (low oxygen concentration), PHD1-3 is inhibited and HIF-1 alpha is not degraded and remains active [20]. When this transcription factor is active, it regulates the expression of several genes; among them, many are proteins involved in glycolysis and lactate pathways, favoring anaerobic ways of obtaining energy and stopping aerobic ones.

It has been shown that, in mouse embryo fibroblasts, HIF-1 directly targets the pyruvate dehydrogenase kinase 1 (PDK1) gene and increases its protein expression [24]. PDK1 inactivates the TCA cycle enzyme pyruvate dehydrogenase (PDH), which is the enzyme responsible for catalyzing the oxidative decarboxylation of pyruvate to acetyl-coenzyme A (the first step in the mitochondrial catabolism of pyruvate). As a consequence, the PDK1 increased expression mediated by HIF-1 slows aerobic metabolism and increases pyruvate levels, and pyruvate will ultimately be converted into lactate. This mechanism is not the only one by which HIF-1-mediated adaptations affect lactate pathways.

Monocarboxylate transporters (MCTs), which are upregulated by high-intensity exercise [25,26,27,28], are further proteins that are affected by HIF-1 alpha mediated adaptations. As described before, MCT4 (Km of 25–31 mM) is the isoform predominant in glycolytic fibers, and MCT1 (Km of 3.5–8.3 mM) is the predominant isoform in oxidative fibers; thus, it is suggested that MCT4 facilitates the release of lactate from glycolytic fibers, and MCT1 facilitates the uptake of lactate in oxidative fibers. Ullah et al. [29] reported that, in cultured cells, HIF-1 mediates lactate transport adaptations by increasing MCT4 protein content and mRNA. Under hypoxia (1% O_2_), MCT4 gene expression and protein content were increased; however, in HIF-1 knock-out cells under the same hypoxic condition, no changes in MCT4 were observed. On the other hand, the same research reported that MCT1 protein expression and contents were not affected by HIF-1 activation.

The regulation of lactate production has also been shown to be indirectly favored by HIF-1 activity. HIF-1 promotes the expression of glycolytic genes that encode the enzyme PFK, the glucose transporters GLUT1-3 in different cultured cells [30,31,32,33], and the GLUT4 translocation in C2C12 myocytes [30]. PFK is the key enzyme of glycolysis and increased levels are associated with higher muscle glycolytic capacity and therefore upregulated lactate production. GLUT1-3-4, which are the glucose transporters in the cell membrane, also favor glycolysis by augmenting glucose transport into the cell. Additionally, in C2C12 myotubes, HIF-1 regulated glycogen synthesis by regulating the expression of the GYS1 gene [34], a gene that is responsible for the synthesis of glycogen synthase. Furthermore, the expression of UGP2 and GBE1 (genes that are in charge of the rest of the enzymes needed for glycogen synthesis) were also induced by hypoxia, but it is still unknown if the expression of these genes is also HIF-1-dependent [34]. It should be noted that the primary limiting factors for glycogen synthesis (apart from substrates availability) is the activity level of glycogen synthase [35], which is upregulated by insulin [36] and glucose 6 phosphate [37], and inhibited by glycogen levels [38]. At the moment, there is no evidence suggesting that a HIF-1-mediated mechanism increases the activity of glycogen synthase.

**Figure 1:**
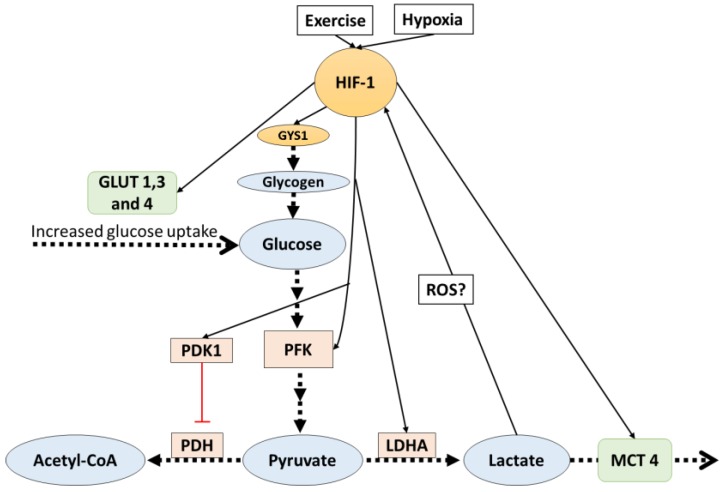
HIF-1 activates signals which favor lactate production. Additionally, lactate indirectly affects HIF-1. The dotted arrows indicate transport and reactions, solid lines indicate increased (black) or inhibit (red) gene expression. HIF-1: hypoxia inducible factor-1; PDK: pyruvate dehydrogenase kinase 1; PFK: phosphofructokinase; PDH: pyruvate dehydrogenase; LDHA: lactate dehydrogenase A; ROS: reactive oxygen species; MCT4: monocarboxylate transporter 4.

Other studies [39,40,41] suggested that the enzyme LDHA (which regulates the conversion of pyruvate to lactate) is regulated in hypoxia by an HIF-1-mediated mechanism, where HIF-1 binds to a LDHA gene promoter, upregulating LDHA production at a transcriptional level. However, it should be noted that LDHA regulation during hypoxia is not only dependent on HIF-1. Despite the fact that HIF-1 activation is essential, it is not sufficient to activate LDHA production; thus, other transcription factor contributions are needed. All the discussed HIF-1-mediated adaptations (Figure 1) lead to increases in the production and removal rates of lactate, and this directly affects the availability of pyruvate for aerobic glycolysis. It has been argued that this may be a mechanism to avoid oxidative stress caused by the increased production of reactive oxygen species (ROS) production from aerobic metabolism, which may be dangerous to cells and is particularly increased during hypoxia [42].

Interestingly, lactate has been shown to activate HIF-1, although none of the studies examined muscle fibers. In endothelial cells cultured with lactate, the expression of HIF-1 and vascular endothelial growth factor (VEGF, which promotes angiogenesis and augments the available surface area for glucose exchange) were upregulated [22,43]. Additionally, in oxidative tumor cells cultured with lactate, HIF-1 was activated [44]; as the used cells were oxidative, this may lead to the hypothesis that ROS produced from lactate catabolism may be mediating HIF-1 activation. Moreover, a study of the same group [22] reported that, in endothelial cells, when MCT1 was blocked, the lactate-induced HIF-1 activation was inhibited. All these data suggest that lactate and HIF-1 activation may have reciprocal control; lactate production is upregulated by HIF-1, and in some way HIF-1 is activated in response to lactate in some cells type. The mechanism behind this reciprocal control is still unclear, but ROS-mediated activation of HIF-1 appears as a possible explanation. This hypothesis is supported by two studies that reported that antioxidants (*N*-acetyl-cysteine, ascorbate and iron) reduced HIF-1 levels in human P493B cells [45] and various human cancer cell lines [46]. It would be of interest in future research to consider a ROS-mediated mechanism by which HIF-1 may be activated to favor anaerobic metabolism and stop ROS production.

## 5. PGC-1 Alpha Mediates Lactate Adaptations in Muscle Fibers

Many adaptations to exercise are mediated by the transcription factor peroxisome proliferator activated receptor gamma coactivator (PGC)-1 alpha. It is widely accepted that PGC-1 alpha is the master controller of mitochondrial biogenesis as well as a regulator of many genes involved in energy metabolism. There is a close relationship between lactate and this transcription factor.

**Figure 2:**
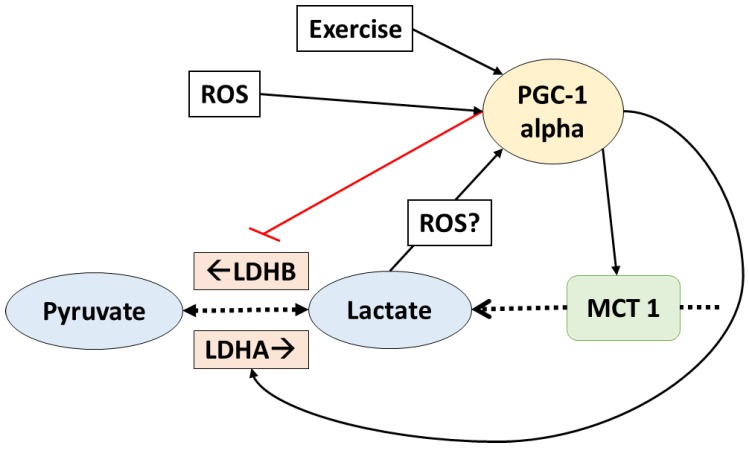
PGC1-alpha controls lactate transport and homeostasis. Lactate also affects PGC1-alpha. The dotted arrows indicate transport and reactions; solid lines indicate increased (black) or inhibit (red) gene expression. ROS: reactive oxygen species; PGC1-alpha: peroxisome proliferator activated receptor gamma coactivator-1 alpha; LDHA/B: lactate dehydrogenase A/B; MCT1: monocarboxylate transporter 1.

In fact, PGC-1 alpha is a key regulator of lactate metabolism. It has been shown that, in C2C12 myotubes, the LDH enzymes are regulated by this transcription factor in a way that favors lactate catabolism [47]. LDHB isoform (which mediates the reaction that produces pyruvate from lactate) is upregulated at a transcriptional level when PGC-1 alpha binds estrogen receptor-alpha on a LDHB promoter. Additionally, the expression of LDHA (enzyme which regulates the conversion of pyruvate to lactate) was downregulated by PGC-1 alpha, apparently by a myelocytomatosis oncogene (Myc) mediated mechanism [47]. Additionally, the expression of MCT1 (which facilitates oxidative fibers lactate uptake) was also suggested to increase in response to PCG-1 alpha, but the mechanism behind this adaptation remains unclear. On the other hand, MCT4 (which facilitates lactate release from the glycolytic fibers) was not affected by PGC-1 alpha in rat muscle cells [48]. All these data suggest that PGC-1 alpha regulates lactate homeostasis by favoring lactate catabolism (Figure 2). It is important to remark that these adaptations were observed in normoxia (i.e., there is enough oxygen to metabolize lactate).

Interestingly, lactate has been shown to upregulate the expression and content of some of the proteins upregulated by PGC-1 alpha. In mice, chronic post exercise lactate consumption increased MCT1 contents in glycolytic fibers [17]. Another study reported that, in L6 cells cultivated with lactate, MCT1, and cytochrome c oxidase complex IV (COXIV), protein context and gene expression were upregulated [49]. Additionally, decreased lactate accumulation in response to chronic dichloroacetate (an activator of PDH) administration reduced the mitochondrial adaptations to high-intensity interval training in mice. Citrate synthetase (CS), beta-HAD, and COXIV as well as the fatty acid transporter FAT/CD36 expression and protein content are increased with high-intensity interval training; however, when dichloroacetate was administrated (and lactate reduced), these adaptations were attenuated [50]. Moreover, PDK isoform 4 (which inhibits the conversion of lactate to pyruvate) and UCP3 (uncoupling protein 3, which improves mitochondrial oxidative capacity) were also increased with lactate administration in mouse muscle fibers—both proteins that are also upregulated by PGC-1 alpha [51].

Furthermore, in L6 cells PGC-1 alpha itself was upregulated when cultured with lactate, which may explain the upregulation of proteins involved in the aerobic metabolism in response to lactate [49]. Moreover, a recent study showed that in vivo lactate administration (by intraperitoneal injection on mice) upregulated PGC-1 alpha gene expression [51]. This clearly suggests that many possible lactate-induced adaptations are mediated by the upregulation of PGC-1 alpha.

Several of the adaptations to high-intensity training are facilitated by PGC-1 alpha, and it is suggested that ROS-mediated mechanisms are involved in PGC-1 alpha regulation [26,52,53,54,55]. In rat skeletal muscle cells, the contraction induced PGC-1 alpha increased expression was not observed with cells incubated with several antioxidants cocktails [54]. Furthermore, another study [55] reported that, in humans and rats, oral supplementation with the antioxidant vitamin C annulated the exercise induced PGC-1 alpha expression (as well as the expression of the mitochondrial biogenesis markers: nuclear respiratory factor 1 and mitochondrial transcription factor 1). At the moment, there seems to be a consensus that lactate-mediated adaptations are produced as a consequence of ROS production and a concomitant effect on PGC-1 alpha pathways, but there is a lack of evidence to confirm this theory yet. Therefore, further research should be conducted to confirm if lactate-induced adaptations are fully mediated by ROS.

## 6. Lactate Stops Other Metabolic Routes

Until now, the discussion has focused on how lactate production is upregulated during hypoxia, and how lactate catabolism is favored during normoxia. However, lactate is also a molecule that is capable of enhancing its own metabolism and inhibiting other metabolic routes, particularly the oxidation of fats.

That lactate inhibits lipolysis was originally reported in 1963 [56]. Lactate infusion in humans attenuated the increase of blood free fatty acids (FFA) and glycerol during exercise [56,57]. The mechanism behind this phenomenon was not understood until recently. Hydroxy-carboxylic acid receptor 1 (HCA1), originally named G protein-coupled receptor (GPR81), is a protein highly expressed in adipose tissue and adipocyte cell membranes. It has been reported that lactate may target and activate HCA1, and that activated HCA1 results in inhibition of lipolysis [58,59]. A lactate threshold needs to be reached for this activation happen. This threshold (5 mmol/L) is on the normal range of lactate levels reached during exercise in humans [58]; therefore, this mechanism has physiological significance. Supporting the anti-lipolytic effects of lactate, lactate infusion has also been shown to decrease plasma catecholamine during exercise [60]. It is well accepted that catecholamine upregulates lipolysis [61], so a decrease in catecholamine levels will reduce lipolysis. All these lactate-induced effects reduce the energy obtained from fats and favor its own metabolism. Other protein expressions affected by lactate in adipocytes include FGF21 [62], which is an activator of glucose uptake in adipocytes [63], and, covertly, UPC1 [64], which may increase the oxidative capacity of adipocytes.

Interestingly, high lactate levels have been suggested to affect glycolysis. PFK is the key enzyme of glycolysis, and its inhibition directly diminishes pyruvate and lactate production. It has been reported, first in C2C12 cells [8] and later in mice [9], that lactate downregulates PFK, in contrast to its effect in hypoxia. The mechanism behind this is still unknown, but this effect directly limits anaerobic metabolism. Further research should be done to establish if this downregulation of PFK is affecting energy production during periods of high energetic demand as exercise. As it was discussed, lactate inhibits lipolysis and has the potential to decrease glycolytic activity; therefore, lactate may be limiting other ways of obtaining energy and indirectly favoring its own metabolism.

## 7. Alternative Signaling Pathways of Lactate

### 7.1. Lactate Anabolic Effects

Research on lactate has not only focused on metabolic related adaptations. It has been suggested that lactate may play a role in muscle cell myogenesis (the process of muscular cell formation). This process particularly takes place within embryo cells, but it is commonly accepted that satellite cells (muscle stem cells) may also differentiate into myocytes (muscle fibers), thus playing an important role in muscle repair, maintenance, and growth [65].

A study conducted by Bloch et al. [66] showed that, in C2C12 myoblast cultured with lactate, myogenesis is affected by a withdrawal from the cell cycle and promotion of early differentiation. However, the addition of an antioxidant (l-ascorbic acid, *N*-Acetyl-l-cysteine and linolenic acid) reversed these lactate-induced effects, suggesting that they are mediated by ROS. Supporting the anabolic lactate effects, a recent study [67] reported that lactate (as well as lactate with caffeine) also promotes differentiation in satellite cells. While lactate increased myogenin protein content (a satellite cell activity marker) and P70S6K phosphorylation (an anabolic marker), lactate with caffeine additionally augmented Pax7, MyoD (satellite cell activity markers), and mTOR phosphorylation (mammalian target of rapamycin; an important anabolism marker). Moreover, in an in vivo study, oral lactate with caffeine supplementation in low exercised rats increased muscle mass, satellite cell activity (myogenin content), and anabolic signals (phosphorylation of mTOR and P70SK) [67]. These results indicate that lactate may also be involved in the activation of anabolic signals.

Another anabolic factor that was related to lactate was the steroid hormone testosterone, which can later activate the mTOR signaling pathway [68,69]. Testosterone secretion was increased in rat Leydig cells after being cultured with 10 mmol of lactate [70]. A similar study [71] with rat testicular cells cultured in lactate (0.01–10 mmol) also reported increases in testosterone production by a luteinizing hormone independent mechanism, suggesting a cAMP-mediated mechanism. The four presented studies are the only ones that relate lactate to testosterone and myogenesis; therefore, further research should be done to confirm these results and to understand how lactate may be affecting satellite cells and muscle growth in living organisms.

### 7.2. Lactate in the Brain

A detailed description of lactate signaling pathways in the brain is a topic that exceeds the scope of this review and has been well reviewed by Mosienko et al. [72]. Nonetheless, this section will briefly summarize lactate functions in the brain. Lactate acts in the brain, both as a signaling molecule and as an energy source. It is produced by astrocytes and neurons, and it has also been suggested that part of the muscle lactate production is shuttled to the brain [73]. Lactate shuttling has been suggested to happen between astrocytes and neurons, like with glycolytic and oxidative fibers. Astrocytes have a high concentration of MCT4, while in neurons the MCT1 isoform is more present; thus, it has been postulated that lactate produced by astrocytes may be used as energy by neurons. Additionally, it was suggested that, during exercise, muscle-derived lactate may supply energy to the brain [74]. Moreover, exercise-mediated adaptations in the brain have been reproduced by oral lactate administration [75]; therefore, it is logical to think that lactate is a signal molecule in the brain.

Cells of the central nervous system possess the lactate receptor HCA1 which is mostly present in neurons, but also expressed in astrocytes. HCA1 is directly activated by lactate, and this causes an inhibition of the cAMP-mediated signaling pathways. Importantly, for HCA1 lactate-mediated activation, a lactate concentration higher than 5 mmol/L is needed [72]. It should be noted that, because the levels of HCA1 in the brain are approximately 100 folds lower than in adipose tissue [76], the relevance of HCA1 in the brain is not clear. Moreover, astrocyte-derived lactate has been shown to be a signaling molecule that mediates excitation of locus coeruleus neurons (which supplies norepinephrine to the frontal brain structures) [77]. This mechanism may have major physiological implications for the nervous system activation.

The role of lactate in the brain has been associated with long-term memory. Suzuki et al. [78] showed that the shuttle of lactate from astrocytes to neurons is necessary to establish and maintain long-term memory in vivo. The molecular mechanism behind this was investigated by Yang et al. [64]. Their study reported that lactate induces the expression of plasticity genes in neurons (Arc, Zif268, c-Fos, and BDNF), and this effect is mediated by the modulation of the NMDA receptor (which is a receptor that regulates neuron plasticity and memory functions). When lactate transport was stopped by a MCTs inhibitor (UK5099), the lactate-mediated effect was no longer observed; thus, lactate transport is necessary for this process. Moreover, the same cell types cultured with NADH (which is produced in the reduction of lactate) simulated the same effects observed in lactate cultured cells; therefore, the lactate-induced plasticity genes expression in neurons is mediated by lactate metabolism and the associated production of NADH (which modify the NADH/NAD ratio, mediating many signaling pathways). It is important to remark that the lactate level needed to activate plasticity-related genes was 2.5 mmol/L. This level is in the range of human brain basal levels, which vary from 0.5 to 5.1 mmol/L [79]; thus, this mechanism has significant relevance in brain plasticity under healthy physiological conditions.

## 8. Conclusions and Future Directions

This review discussed the several pathways that lactate may have. Lactate to pyruvate reversing conversion is of fundamental importance to maintain the redox balance in cells. Furthermore, lactate has many benefits during exercise: lactate transport serves as an indirect regulator of muscle acidosis, and lactate itself is an effective energy source. As a signaling molecule, lactate has been shown to interact with many important pathways, such as the case of HIF-1, PGC-1, alpha, and many other anabolic signals. Additionally, lactate function in the brain is far from being understood; however, apart from being an energy source, it is implicated in important functions such as norepinephrine release and brain plasticity. Lactate is one of many molecules that are increased during exercise, and it is highly transported between tissues, having many different roles. Despite its clear influence in many signaling pathways, it is not clear yet how lactate affects these pathways; therefore, further research must be done to clarify the mechanism by which lactate is mediating adaptations, particularly exercise-induced adaptations. Additionally, due to the apparently positive effects (exercise-like induced adaptations) of lactate in the organism, it would be interesting to consider it as a sports supplement. Although there are some studies that report lactate as an ergogenic aid presented as an energy source as well as an alkalizing agent [80,81], there is no evidence on how oral lactate supplementation can affect exercise-mediated adaptations in humans. Furthermore, it would be of interest to study the effects of lactobacillus fermented food as a natural source of lactate. In addition, despite the acceptance of the theory that lactate mediates exercise-induced adaptations, it is unknown if it has the same effect in both trained and untrained subjects. These are examples of questions that are still unsolved. Lactate science is far from being completely understood; therefore, it is still an exciting area to study.
